# Bidirectional cross-day alignment of neural spikes and behavior using a hybrid SNN-ANN algorithm

**DOI:** 10.3389/fnins.2026.1772958

**Published:** 2026-02-04

**Authors:** Binjie Hong, Zihang Xu, Tengyu Zhang, Tielin Zhang

**Affiliations:** 1 Center for Excellence in Brain Science and Intelligence Technology, State Key Laboratory of Brain Cognition and Brain-inspired Intelligence Technology, Institute of Neuroscience, Chinese Academy of Sciences, Shanghai, China; 2 Brain Science Data Center, Chinese Academy of Sciences, Shanghai, China; 3 Beijing Forestry University, Beijing, China

**Keywords:** brain activity simulation, brain-computer interface, contrastive learning, neural decoding, spiking neural network

## Abstract

Recent advances in deep learning have enabled effective interpretation of neural activity patterns from electroencephalogram signals; however, challenges persist in invasive brain signals for cross-day neural decoding and simulation tasks. The inherent non-stationarity of neural dynamics and representational drift across recording sessions fundamentally limit the generalization capabilities of existing approaches. We present AlignNet, a novel framework that establishes cross-modal alignment between spiking patterns and behavioral semantics through U-based representation learning. Our architecture employs hybrid SNN-ANN autoencoders to encode neural spikes and behavior into a shared latent space, where the neural spike autoencoder incorporates multiple neuron nodes following convolution layers, and the behavior autoencoder comprises standard convolution layers. These two representations are optimized through contrastive objectives to achieve session-invariant feature learning. To address cross-day adaptation challenges, we introduce a pretraining strategy leveraging multi-session single monkey experiment data, followed by task-specific fine-tuning for neural decoding and simulation. Comprehensive evaluations demonstrate that AlignNet achieves superior performance under both single-day and cross-day conditions; meanwhile, our pretrained model effectively executes decoding and simulation tasks after fine-tuning. The hybrid SNN-ANN representations exhibit temporal consistency across multi-day recording spikes while retaining behavioral semantics, thereby advancing cross-day neural interface applications.

## 1 Introduction

In the field of neuroscience, the decoding of neural data has been advanced by deep learning techniques. Animal experiments provide compelling evidence that behavioral states are strongly correlated with specific patterns of brain activity. Research demonstrates that dynamic firing-rate modulations critically influence both the initiation and suppression of motor behaviors (Falasconi et al., 2025). Deciphering the complex information embedded in neural signals represents a fundamental challenge at the intersection of neuroscience and computational science. To address this challenge, novel computational frameworks have been developed. Azabou et al. (2023) introduce POYO, a transformer-based architecture that tokenizes neural spike data and utilizes cross-attention to model population-level dynamics across extensive neural recordings. Researchers (Vermani et al., 2023) propose an unsupervised alignment method designed to leverage shared latent dynamics, facilitating the transfer and reuse of pre-trained generative models across diverse neural datasets.

However, neural decoding encounters challenges in cross-day sessions. Neural non-stationarity (Gowreesunker et al., 2011) and factors such as postoperative inflammation (Giunta et al., 2024) induce drift in the behavior-neural signal relationship, compromising decoding stability. To address it, cross-day neural decoding frameworks have been developed. For instance, the Neural Speech Decoding Framework (Chen X. et al., 2024) employs pretrained synthesizers to map neural features to speech waveforms, while domain-invariant representation learning aligns feature distributions across sessions. Neural Encoding Dynamic Sampling (NEDS) (Du et al., 2023) constructs a meta-representation space that captures stable neural dynamics. It employs central kernel alignment (CKA) to quantify cross-day similarity and dynamically calibrates decoder parameters.

Beyond mapping neural data to behavioral outputs, simulating biologically realistic neural dynamics remains a significant yet underexplored challenge in computational neuroscience. To address this gap, Aldarondo et al. (2024) designed an artificial neural network that simulates rodent cortical dynamics by integrating multi-scale biological constraints, including spike-timing-dependent plasticity and laminar-specific connectivity pattern. miVAE (Zhu et al., 2025) proposed a two-stage disentanglement framework that jointly maps neural activity and sensory stimuli into a unified latent space. This approach enables robust identification of cross-modal correlations without requiring subject-specific calibration.

We propose a hybrid SNN-ANN framework that integrates autoencoders and contrastive learning module to complete simultaneous behavioral decoding and neural spike encoding. The proposed architecture leverages the temporal dynamics of spiking neural networks (SNNs) to effectively extract features from neural spike data, while employing artificial neural networks (ANNs) to capture rich representations from corresponding behavioral data. We combine the feature extraction capabilities of U-shaped models for motion trajectories and neural signals with the generalization strengths of contrastive learning. The incorporation of spiking neural networks contributes to adopt biological mechanism when encoding and decoding natural spikes. Evaluation results demonstrate that our framework outperforms existing methods not only in single-day tasks but also in cross-day scenarios.

The main contributions of our work are summarized as follows:

- **Bidirectional alignment framework**: A novel approach for joint embedding of neural spikes and behavior via similarity constraints across trials. It enables discovery of a latent space where stable neural manifold and behavior are bidirectional aligned.
- **Hybrid SNN-ANN autoencoders**: Integration of SNNs (capturing biological fidelity) for robust reconstruction of neural data and ANNs for sequential behavior data utilizes distinct properties of two modalities.
- **Cross-day generalized capability**: A pretraining protocol optimizes on multi-days animal experimental datasets, enhancing generalization of understanding neural signals and corresponding behaviors. The comparison experiments show SOTA ability after finetuning the model on spike-to-behavior decoding and behavior-to-spike simulation tasks.

## 2 Related works

### 2.1 Neural signal decoding and simulation

Brain-computer interface (BCI) technology represents a transformative paradigm in human-machine interaction. It enables direct communication between the brain and external devices without relying on conventional neuromuscular pathways (Tangermann et al., 2012; Song et al., 2025). BCI system acquires and decodes neural signals to translate user intentions into actionable commands, generating applied fields, including neurorehabilitation (Zhang et al., 2024), assistive technologies (Awuah et al., 2024), cognitive augmentation (Khorev et al., 2024) and immersive communication (Ghasemi et al., 2024). These technique delineates the operational framework and implementation guidelines for BCI protocols, with emphasis on signal acquisition (Sun et al., 2025) and neural decoding (Dong et al., 2023).

Signal acquisition constitutes the foundational stage of the BCI system, directly determining the quality and interpretability of subsequent neural data analysis. This process involves the recording of electrophysiological or hemodynamic activity from the brain using either non-invasive modalities, including electroencephalography (EEG) (Binnie and Prior, 1994), functional near-infrared spectroscopy (fNIRS) (Naseer and Hong, 2015) and magnetoencephalography (MEG) (Proudfoot et al., 2014), or invasive approaches like electrocorticography (ECoG) (Farias et al., 2008) and intracortical microelectrode (Wang et al., 2023) arrays. Key technical considerations include electrode placement and density, sampling rate, signal-to-noise ratio optimization, artifact suppression, and the selection of appropriate signal paradigms.

Decoding neural signals into behavioral outputs has developed significant progress in current research. POYO (Azabou et al., 2023) model introduces transformer framework to enable efficient training across multiple sessions of neural recordings, which advancing the decoding applications. A recent study by (Cao et al., 2025) demonstrated that recording just a small part of the total neurons from dorsal premotor cortex can help decode 3D hand movement trajectories with high accuracy. The BraVL (Du et al., 2023) model aligns brain activity, visual images, and textual descriptions in a shared latent space, achieving a high decoding accuracy on untrained new categories. It demonstrates the strong generalization capability of contrastive learning in neural decoding tasks.

Although recent advances in algorithms have demonstrated their potential for neural decoding, cross-day neural decoding remains a challenge due to neural signal shift and behavior state variability (Bashford et al., 2024). To solve this problem, researchers worked in different aspects. A new neural speech decoding framework that combines ECoG signal processing with deep learning improves the result of cross-day decoding (Chen X. et al., 2024). Researchers also designed the NEDS (Zhang et al., 2025b) network to help improve encoding and decoding efficiency at the same time. This network leverages multitask masking, alternating between neural, behavioral, and cross-modal masking during training. This method is able to predict behavior from neural activity and predict neural activity from behavior at the same time. Unlike NEDS, we employ a couple Unet-based architecture combined with contrastive learning to enhance spike-to-behavior accuracy.

Simulating neural activity from a sequence of behavior is a significant study in neuroscience, and breakthroughs in this direction could substantially advance the field of BCIs. Recent work has shown that reinforcement learning can mimic the behavior of freely moving rats and predict neural activity in the sensorimotor striatum and motor cortex (Aldarondo et al., 2024). Researcher developed a neuromorphic tactile system that uses spike timing, especially first-spike timing, to encode dynamic tactile information about touch and grasp (Chen L. et al., 2024). Furthermore, the NEUSPA modal (Yan et al., 2025) successfully integrates Izhikevich neurons with motor unit differentiation to simulate abnormal spike activity in post-stroke spasticity. Moreover, miVAE (Zhu et al., 2025) employs a two-level disentanglement strategy to map neural activity and visual stimuli into a unified latent space with artifical neural networks(ANN). Compared with miVAE, our hybrid SNN-ANN model incorporates bidirectional alignment to discover neural manifold and build shared latent space; meanwhile, pretraining protocol boost the generalized capability of cross-day spike-to-behavior decoding and behavior-to-spike simulation.

### 2.2 Deep learning models in BCI

CNNs have been widely applied in neural signal decoding research. For instance, researchers at Purdue University employed a CNN-based framework to implement neural encoding and decoding for dynamic natural vision (Wen et al., 2018). In a parallel development, a hybrid CNN-Transformer architecture was introduced by a research group from the University of Electronic Science and Technology of China (Wen et al., 2018), demonstrating enhanced efficiency in decoding visual neural activity into textual descriptions. Furthermore, the CSM model (Petrosyan et al., 2021) successfully decoded macaque finger movement trajectories by incorporating temporal dependencies through CNN structures, effectively correlating current neural states with preceding moments.

Spiking neural networks (SNNs) are biologically-inspired neural networks Zhang et al. (2023); Wei et al. (2024) that process information through discrete, asynchronous spikes or action potentials, stimulating the event-driven communication of biological neurons. Compared to ANNs, SNNs work better in event-driven data (Deng et al., 2020) and perform efficiently in computer vision tasks Zhang et al. (2021b). CREST (Mao et al., 2025) is an event-based object detection framework. It leverages model with an attention-based bridge, converting spikes to dense features while preserving spatiotemporal dynamics. With lower energy cost, the Spike-Driven Transformer incorporates binary spike communication and spike-driven self-attention (SDSA), achieving high accuracy on the ImageNet-1K dataset (Yao et al., 2023).

Inspired by the biological dynamics of neurons, spiking neural networks have emerged as a pivotal model in computer vision and neuroscience fields Yao et al. (2024). For example, Spike Voxel Coding (SVC) (Qiu et al., 2025) model utilize SNNs network to solve various 3D computer vision tasks, including classification, detection and segmentation. Recent advances in the Dual-Spike Self-Attention (DSSA)mechanism (Shi et al., 2024) and the Self-Backpropagation of Synaptic Modifications (SBP) (Zhang et al., 2021a) enhance performance of the image classification task. The adaptive firing neuron model (AdaFire) also works well in event-driven classification, object detection, and segmentation tasks (Wang et al., 2025). Apart from the work in computer vision tasks, the spiking-based networks have proven to work well to bridge the gap between neuroscience and behavior. The motorSRNN (Liu et al., 2024) and STAA-SNN (Zhang et al., 2025a) both prove the high accuracy and efficiency of decoding from electrophysiology to tasks. Moreover, the spiking-based transformers outperform in event-stream tasks (Yao et al., 2021) and efficient training process (Yao et al., 2025).

In the field of representation alignment, contrastive learning has emerged as a dominant approach, achieving remarkable performance gains across language (Pan et al., 2021), vision (Yang et al., 2022) and multimodal domains (Liu et al., 2023). However, conventional methodologies remain critically dependent on large batches of negative samples, introducing substantial computational overhead and inducing representation bias (Zolfaghari et al., 2021). Recent advances mitigate these limitations through asymmetric network architectures and cross-modal alignment techniques (Jiang et al., 2025), enhancing the efficacy of unsupervised paradigms. CLIP (Li et al., 2021) and FLAVA (Singh et al., 2022) utilize dual-stream encoders to align vision-language embeddings. Subsequent work like CoCa (Yu et al., 2022) unifies contrastive and generative objectives, outperforming supervised baselines across downstream tasks.

## 3 Methods

### 3.1 Dataset description and preprocessing

**Figure 1:**
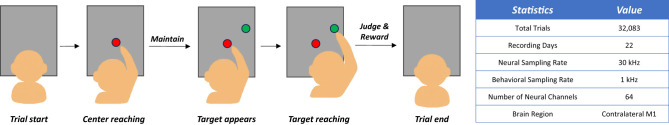
Center-out task and data statistics. The left subplot demonstrates the pipeline of one trial of center-out task. This animal experiment records how a macaque to react when the target appears and the brain activity is recorded simultaneously. The right subplot shows statistics of our dataset.

In this study, the dataset of Joystick-controlled robotic arm whack-a-mole data from a macaque was obtained from the Brain Science Data Center, Chinese Academy of Sciences^1^, where the open access data repository are published. All animal procedures were conducted in accordance with protocols approved by the Institutional Animal Care and Use Committee (IACUC) of the Institute of Neuroscience, Center for Excellence in Brain Science and Intelligence Technology, Chinese Academy of Sciences. During the experimental procedure, neural activity was recorded from a macaque performing an eight-direction center-out task, as illustrated in Figure 1. Signals were collected from the macaque's contralateral primary motor cortex (M1). The dataset comprises neural signals acquired at 30 kHz across 64 channels, along with simultaneous 1 kHz two-dimensional joystick voltage signals that serve as the decoding ground truth. Our dataset comprised 32,083 trials collected over 22 days, with neural signals and macaque behavioral signals simultaneously recorded during the experimental procedure. All neural recordings were obtained from a macaque monkey during continuous sessions between April 1, 2025, and April 29, 2025.

**Figure 2:**
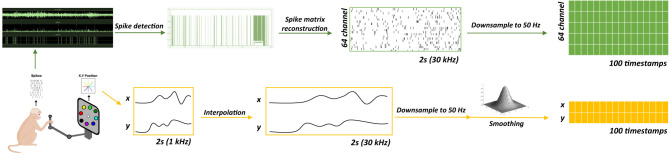
Data preprocessing procedure. The experiment mainly contains four steps including data collection, spike detection, data reconstruction and downsampling. After preprocessing steps, neural spike data will be segmented into 64 channels with 100 sampling points while behavioral data will be reshaped into 2 channels with 100 sampling points.

The preprocessing pipeline, illustrated in Figure 2, involved the following steps: spike detection, spike matrix reconstruction, behavioral data interpolation, downsampling, and smoothing. Neural preprocessing commenced with spike detection on the raw 64-channel recordings to identify action potentials, converting the continuous data into discrete spike events to improve subsequent feature extraction. Subsequently, a spike matrix was reconstructed by aligning these waveforms, extracting their features, and structuring them into a two-dimensional matrix. The x-axis represents the timestamp, and the y-axis corresponds to the channels, with a sampling rate of 30 kHz for both the spike matrix and behavioral data.

To achieve temporal synchronization, behavioral data were interpolated to match the sampling rate of the neural data, followed by spike matrix reconstruction. The linear interpolation function reshapes the behavioral data to the same sampling rate as the spike data, as described in Equation 1,


$$\begin{array}{l} y\left[j\right]=\left(1-\alpha \right)\cdot x\left[\left\lfloor p_{j}\right\rfloor \right]+\alpha \cdot x\left[\left\lceil p_{j}\right\rceil \right]. \end{array}$$ (1)

In this equation, $p_{j}=j\cdot \frac{m-1}{n-1},\;n=0,1,\ldots ,n-1$, α = *p*_*j*_ − ⌊*p*_*j*_⌋. *m* and *n* denotes the total number of sampling points for raw signal and resampled signal, *p*_*j*_ represents the position of j-th sample in the original signal that corresponds to the j-th sample of the target signal. *x*[⌊*p*_*j*_⌋] and *x*[⌈*p*_*j*_⌉] denote the lower and higher index of sampling point in the raw signal near the resampled point.

Both neural and behavioral data were then downsampled to a unified sampling rate of 50 Hz. The downsampling equation for neural spikes is given in Equation 2


$$\begin{array}{l} P_{k}=\overset{\left(k+1\right)I-1}{\underset{t=kI}{\sum }}P\left[t\right],\; \end{array}$$ (2)

where *P*_*k*_ denotes spike, k represents the trial number and I indicates the window width. For the behavior signal, the downsampling equation is implemented similarly to the interpolation step, as shown in Equation 1. After aligning the spike and behavior data, a smoothing filter was applied to the behavioral data to enhance the signal-to-noise ratio (SNR). The equation for the Gaussian noise filter is provided in Equation 3


$$\begin{array}{l} G\left[i\right]=\frac{1}{\sqrt{2\pi }\sigma }\exp\left(-\frac{i^{2}}{2\sigma^{2}}\right),\;i=-k,-k+1,\ldots ,0,\ldots ,k-1,k, \end{array}$$ (3)

where σ represents the standard deviation, i denotes the i-th sampling points in the signal. The Gaussian smoothing filter reduces noise and high-frequency components while preserving the overall signal shape through weighted averaging with Gaussian weights, thereby better capturing the macaque's behavior.

After the aforementioned preprocessing steps, the final preprocessed data structure comprised a 64-dimensional spike train and a 2-dimensional trajectory per trial. Each trial was segmented into 2-second samples containing 100 temporal points before being input to the network.

### 3.2 Proposed method

#### 3.2.1 Overall architecture

**Figure 3:**
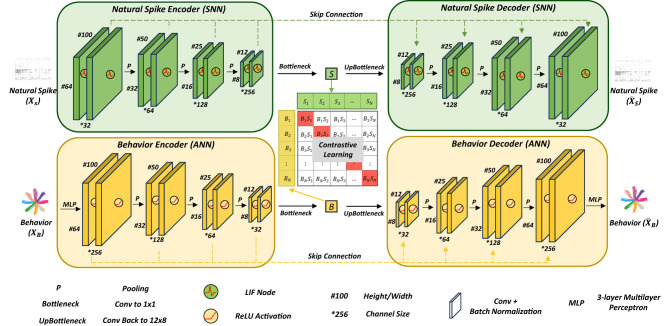
Hybrid SNN-ANN pretrain architecture. The model consists of three core modules: natural spike autoencoder (green), behavior autoencoder (yellow), and contrastive learning module (red). During the process of encoding, neural data and behavior are embedded through multiple convolution module, with batch normalization, activation layer and pooling operation. In the decoding phase, the model utilizes latent representations and skip connection to reconstruct the original input. In t2he autoencoder of natural spike, the activation layer is a Leaky Integrate-and-Fire node while ReLU activation function is utilized in the behavior autoencoder. To achieve cross-modal alignment between neural activity and behavioral patterns, contrastive learning constrains the latent representations generated by both autoencoders within a shared latent space.

We propose AlignNet, a novel framework for contrastive learning of alignment of spike-and-behavior patterns. The architecture comprises three core components: a SNN-based autoencoder dedicated to natural spike data, an ANN-based auencoder for behavior data, and a contrastive learning module. Figure 3 illustrates the overview of the AlignNet framework.

The autoencoder performs unsupervised learning by learning an efficient representation (encoding) of input data through training the network to accurately reconstruct its original input. In AlignNet, two autoencoders with unsharable weights are employed to reconstruct natural spikes and behavior. We set U-based network as the basic framework for our dataset, the multi-layer convolutions extract the features of spikes and behavior temporally and spatially; meanwhile, skip connection ensures information reusage for the process of upsampling and deconvolution. Instead of conventional ANNs, we utilize SNNs for encoding and decoding brain activities since such spiking-based networks model neural dynamics more closely, typically composed of multiple layers of spiking neurons. Specifically, in our implementation, we adopt the Leaky Integrate-and-Fire (LIF) spiking neuron nodes (Maass, 1997), which will be illustrated in detail later.

The contrastive learning component facilitates the construction of a shared latent space for both natural spikes and behavior, where each data modality is projected into hidden vector representations. This technique operates by contrasting similar (positive) and dissimilar (negative) data samples. Rather than relying on explicit labels, it leverages the inherent structure of the data to learn discriminative features and encourage alignment between the spike and behavior representations. By integrating contrastive learning, the representations of natural spikes and behavior become constrained within a coherent and aligned latent space.

#### 3.2.2 Hybrid SNN-ANN autoencoders

Behavioral experiments involved simultaneous recordings of natural spiking activity and corresponding behavioral sequences during a Center-Out task. In this task, monkeys guided a cursor via a joystick to interact with visual targets. The neural data consisted of spiking signals, while the behavioral data were quantified as the trajectories of the cursor movements across trials. The encoder-decoder framework is grounded in the principle of reconstructing input data from its latent representations. The fundamental operations are defined as:


$$\begin{array}{l} \text{z}=\text{f}\left(\text{x}\right), \end{array}$$ (4)

$$\begin{array}{l} \hat{\text{x}}=\text{g}\left(\text{z}\right). \end{array}$$ (5)

Specifically, the encoder *f*(·) transforms the input data x into a lower-dimensional latent representation z, and the decoder *g*(·) subsequently aims to reconstruct the original input from z.

To facilitate contrastive learning in our framework, the latent vectors derived from both spike and behavior data must share the same dimensionality. We therefore employed a Multi-Layer Perceptron (MLP) to project the behavioral trials into the same dimensional space as the neural dSata prior to the encoding step. This design also aids the U-based encoder in more effectively capturing the temporal dynamics present in both the spiking and behavioral embeddings. As the research objective is to achieve bidirectional mapping–decoding behavior from spikes and simulating spikes based on behavior–we adopted a Mean Squared Error (MSE) loss function to train the U-based Autoencoders.

The encoder architecture sequentially integrates Convolution, Batch Normalization, ReLU activation, and Pooling operations. Spiking data consists of non-negative, multi-channel voltage signals that exhibit temporal variations, capturing diverse neural activities across different brain regions. We utilize a U-based encoder to process trials of natural spike data ($X_{S}\in {\mathbb{R}}^{S\times C}$), enabling the extraction of both relative temporal patterns within time intervals (*S*) and spatial correlations across channels (*C*). The mathematical representation of natural spike encoding is defined as follows:


$$\begin{array}{l} H_{spike}=\text{Bottleneck}\left({\text{Downsample}}^{*k}\left(X_{S}\right)\right), \end{array}$$ (6)

$$\begin{array}{l} \text{Bottleneck}=\text{LN}(W^{1}*\text{AvgPool}(X))), \end{array}$$ (7)

$$\begin{array}{l} \text{Downsample}=\text{MaxPool}\left(\text{CONV}\left(X\right)\right), \end{array}$$ (8)

$$\begin{array}{l} \text{CONV}=\text{LN}\left(BN\left(W^{3}*\text{LN}\left(BN\left(W^{2}*X\right)\right)\right)\right), \end{array}$$ (9)

where $H_{Spikes}\in {\mathbb{R}}^{S\times D}$ is the hidden vector of nature spikes and Bottleneck reshapes the output of *k*-layer downsample into *D*-dimension vectors. Specifically, the bottleneck process includes AvgPool denoting 1x1 average pooling with stride 1, *W*^1^ representing an 1 × 1 convolution kernel and an ReLU activation function (max(0, *x*)). Downsample contains a convolution stage and a function of MaxPool denoting 2 × 2 max pooling with stride 2. In the convolution layer, * represents convolution operation and *W*^(*i*)^ are 3 × 3 convolution kernels with distinct input and output dimensions. Moreover, *BN* represents batch normalization and LN denotes the LIF neuron that can be described as:


$$\begin{array}{l} V^{t,n}=H^{t-1,n}+\frac{1}{\tau }\left[I^{t-1,n}-\left(H^{t-1,n}-V_{\text{reset}}\right)\right], \end{array}$$ (10)

$$\begin{array}{l} S^{t,n}=\Theta \left(V^{t,n}-v_{\text{th}}\right), \end{array}$$ (11)

$$\begin{array}{l} H^{t,n}=V_{\text{reset}}\cdot S^{t,n}+V^{t,n}⊙\left(1-S^{t,n}\right), \end{array}$$ (12)

where τ is the membrane potential time constant, *t* and *n* respectively denote the indices of the time step and the n-th layer. *H*^*t* − 1, *n*^ is the membrane potential from previous time step, *I*^*t, n*^ denotes the input data at the time step *t* and the *n*-th layer, and *V*^*t, n*^ is the updated membrane potential. *S*^*t, n*^ is the spike sequence through the Heaviside function Θ, in which *v*_*th*_ determines whether *V*^*t, n*^ triggers a spike or remains silence. In our research, we fix τ, *v*_*th*_ and *V*_reset_ as 2, 0.3 and 0 respectively.

Similarly, the process of encoding behavior from a sequence of actions ($X_{B}\in {\mathbb{R}}^{S\times 2}$) to hidden vectors ($H_{Behavior}\in {\mathbb{R}}^{S\times D}$) works through *k*-layer downsample and botteneck. Different with natural spike encoding, LNs are replaced with σ denoting ReLU activation function(max(0, *x*)).

The decoder is trained to reconstruct input data from latent representations. Both natural spike and behavior decoders consist of 4 steps including Upsampling, Convolution, Batch Normalization and activation. Skip connection is used for creating direct pathways between encoder and decoder layers by concatenating feature maps from the encoding path with corresponding upsampled features in the decoding path. Therefore, the decoder is defined as:


$$\begin{array}{l} {\hat{X}}_{spike}={\text{Upsample}}^{k}\left(H_{spike}\right), \end{array}$$ (13)

$$\begin{array}{l} \text{Upsample}=\text{LN}\left(BN\left(W^{\left(2\right)}*\text{LN}\left(BN\left(W^{\left(1\right)}*X^{\text{cat}}\right)\right)\right)\right), \end{array}$$ (14)

$$\begin{array}{l} X^{\text{cat}}=\text{Concat}\left(X^{\text{downsample}},H_{spike}\right), \end{array}$$ (15)

where Upsample denotes *k*-layer upsampling (transposed convolution, Batch Normalization and activation), *X*^downsample^ is the corresponding encoder-level feature map (skip connection).

#### 3.2.3 Contrastive learning

In AlignNet, contrastive learning paradigm learns representations of behavior and simulating natural spikes by contrasting positive pairs against negative pairs in a latent space. Given an anchor sample, the target is to pull positive samples closer while pushing negative samples farther apart. The negative sample in this study is defined as the non-responding behavior or nature spikes out of one trial. According to the task of behavior decoding and natural spike simulation, multimodal domains are obejective to be aligned. Therefore, we levarage a batch of *N* spike-behavior pairs ${\left\{\left(S_{i},B_{i}\right)\right\}}_{i=1}^{N}$, the contrastive learning objective is to reduce the loss of:


$$\begin{array}{l} L_{\text{CL}}=\frac{1}{2}\left(L_{Spike}+L_{Behavior}\right), \end{array}$$ (16)

where the spike-to-behavior loss is:


$$\begin{array}{l} L_{Spike}=-\frac{1}{N}\overset{N}{\underset{i=1}{\sum }}\log\frac{\exp\left(\langle f_{s}\left(X_{S}\right),f_{b}\left(X_{B}\right)\rangle /\tau \right)}{\sum_{j=1}^{N}\exp\left(\langle f_{s}\left(X_{S}\right),f_{b},\left(X_{B}\right)\rangle /\tau \right)} \end{array}$$ (17)

and the behavior-to-spike loss $L_{Behavior}$ is symmetrically defined. *f*(·) is an embedding function corresponds natural spike encoder or behavior encoder in our framework. τ is a temperature hyperparameter. We constrict the hidden vectors in the latent space by maximizing the similarity of the spike-and-behavior pairs. Consequently, the embedding of natural spikes can be considered as the represatation of behavior, which is decoded into behavior; similarly, the embedding of behavior can be decoded by the natural spike decoder directly to simulate the brain activity.

## 4 Experiments and results

### 4.1 Training setting

In the experimental design, the dataset was partitioned into three distinct subsets: pretraining, fine-tuning, and testing. The pretraining phase involves joint optimization of the encoder and decoder to model both neural spiking dynamics and behavioral patterns. Data from 19 consecutive days were allocated for pretraining, during which neural and behavioral representations are learned within a shared latent space to achieve cross-day alignment. This pretrained model is expected to exhibit enhanced generalization capability across different recording days, thereby improving overall model efficiency.

**Figure 4:**
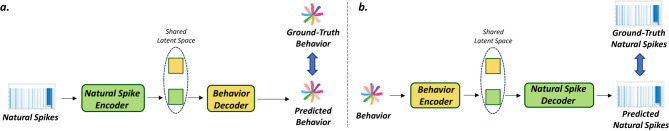
Strategies of fine-tuning tasks (Neural decoding and simulation). Arrows denote supervised training signal flow. During fine-tuning, input spike embeddings are regarded as latent proxies of ground truth, leveraging contrastively-aligned representations. **(a)** Neural decoding. **(b)** Neural simulation.

During the fine-tuning stage, we conducted two downstream tasks: neural decoding and natural spike simulation, where all parameters were fine-tuned. Implementation strategies for both tasks are detailed in Figure 4. During the fine-tuning stage, we implement a supervised training paradigm where target labels are guided to predict behavioral trajectories or natural spike patterns. For evaluation, input and output representations are regarded as aligned in the embedding space, since the model's contrastive pretraining establishes consistent latent representations for behavioral and neural dynamics. Consequently, natural spikes are encoded into latent representations via the spike encoder, followed by fine-tuning of the parameters in the behavior decoder to obtain enhanced prediction results.

To evaluate cross-day generalization, we established two experimental protocols: single-day and cross-day. For the single-day task, we split 80% of the data from one day as training set and 20% from on the same day as evaluation set. In the cross-day task, two days of data was used for training and one held-out day for evaluation, assessing generalization capability.

### 4.2 Performance evaluation

To evaluate performance of our model, we benchmark the model performance against several established models to ensure a comprehensive comparison. In commonly used models, we employ the Wiener Filter, Gated Recurrent Unit (GRU), and vanilla MLP as benchmarks. The Wiener Filter (Glaser et al., 2020) represents the classical statistical approach, providing an optimal linear estimate under stationary assumptions. For deep learning baselines, we include GRU model to handle the time-series nature of the data and a MLP as a simple yet powerful feed-forward benchmark. The GRU's ability to learn from historical information makes it suitable for modeling the temporal dynamics of neural activity, while the MLP tests the efficacy of treating sequences as fixed-dimensional vectors. This selection allows us to contrast linear, recurrent, and dense non-linear methodologies.

Beyond these comparatively simple benchmarks, we delve into more complex architectures to implement decoding and simulation. Besides UNet structure, we tried to utilize Resnet, which enables the training of extremely deep networks by mitigating the vanishing gradient problem, allowing gradients to flow directly through these identity mappings. Building on this, a hybrid Resnet+UNet model is implemented, where the Resnet encoder captures features at multiple scales, and the UNet decoder pathway facilitates precise, detailed output generation by combining high- and low-level features, which is critical for accurate signal reconstruction.

In our experiments, the evaluation metrics primarily include R-square Error and Pearson correlation coefficient(PCC). R-square Error serves as a key measure for assessing regression performance by quantifying the proportion of variance explained by the model. The equation of R-squared function can be shown as Equation 18.


$$\begin{array}{l} R^{2}=1-\frac{\sum_{i=1}^{n}{\left(y_{i}-{\hat{y}}_{i}\right)}^{2}}{\sum_{i=1}^{n}{\left(y_{i}-\bar{y}\right)}^{2}}, \end{array}$$ (18)

where *y*_*i*_ means the actual value of the i-th observation, ŷ_*i*_ means the predicted value of the i-th observation, ȳ means the mean of actual values.

The PCC evaluates the linear correlation between predicted movements and ground-truth data, with its calculation involving covariance normalization; this metric is also employed in spike simulation tasks to measure the similarity between simulated and natural brain activity patterns, where higher values indicate greater pattern similarity. The equation can be shown as


$$\begin{array}{l} \text{PCC}=\frac{\sum_{i=1}^{n}\left(x_{i}-\bar{x}\right)\left(y_{i}-\bar{y}\right)}{\sqrt{\sum_{i=1}^{n}{\left(x_{i}-\bar{x}\right)}^{2}}\sqrt{\sum_{i=1}^{n}{\left(y_{i}-\bar{y}\right)}^{2}}}. \end{array}$$ (19)

In this equation, *x*_*i*_ and *y*_*i*_ means the value of the i-th observation for variable X and Y while $\bar{x}$ and ȳ means the sample mean of variable X and Y.

### 4.3 Results

The performance of various models on spike-behavior and behavior-spike prediction tasks is summarized in Table 1. We conducted both single-day and cross-day experiments to evaluate the proposed model's decoding capability and generalization performance.

**Table 1:** The performance of different models under single-day and cross-day conditions.

**Models**	**Single-day**		**Cross-day**	
	**spike → behavior(*R*^2^)**	**behavior → spike(PCC)**	**spike → behavior(*R*^2^)**	**behavior → spike(PCC)**
Wiener Filter (Glaser et al., 2020)	0.354	0.442	0.08	0.409
GRU	0.250	0.194	0.09	0.204
MLP	0.723	0.492	0.366	0.349
Resnet	0.413	0.323	0.327	0.201
Resnet+UNet	0.327	0.227	0.390	0.112
Ours	0.880	0.539	0.581	0.440

#### 4.3.1 Single-Day performance

In the single-day experiments, the models were trained and tested on data from the same recording day. Eighty percent of the data were used for training and the remaining twenty percent for testing. This experiment was designed to evaluate the model's ability to learn the relationship between spike activity and behavioral features within one day.

Traditional models, the Wiener filter and GRU, exhibited limited predictive capability. The Wiener filter achieved an R^2^ of only 0.354 for spike-behavior decoding and a PCC of 0.442 for behavior-spike prediction. The GRU performed even worse, with an R^2^ of 0.250 and a PCC of 0.194. These results indicate that such models struggle to capture the nonlinear and dynamic relationship between neural signals and behavior. The MLP model performed slightly better, with an R^2^ value of 0.723 for spike-behavior prediction and a PCC of 0.492 for behavior-spike prediction. However, its performance is still limited by its insufficient ability to capture the complex dynamics of neural signals.

Our proposed model significantly outperformed these baselines. It achieved an R^2^ of 0.880 for spike-behavior decoding and a PCC of 0.539 for behavior-spike prediction. This improvement can be attributed to the model's integrated U-Net architecture, contrastive learning mechanism, and bidirectional design, which together enable more robust feature extraction and cross-modal alignment. Furthermore, after a single training session, our model can directly perform bidirectional reasoning through fine-tuning. It can adapt to different task requirements without changing the model architecture. This unified framework significantly enhances the model's versatility and deployment efficiency. In contrast, other models usually need to adjust their architectures when dealing with different tasks, which increases the complexity of development and maintenance.

#### 4.3.2 Cross-Day performance

To evaluate model generalization across time, we further conducted cross-day experiments. In this setting, the models were trained on data from two consecutive days and tested on data from a third day. The goal was to test whether models can adapt to new data recorded on different days, where neural activity and behavior may vary.

Traditional models showed a clear degradation in performance. The Wiener filter achieved an R^2^ of only 0.08 for spike-behavior decoding and a PCC of 0.409 for behavior-spike prediction. The GRU model performed similarly poorly, with an R^2^ of 0.09 and a PCC of 0.204. These results indicate that the traditional model has significant deficiencies in its cross-day generalization ability. The deep learning models MLP and ResNet + UNet performed slightly better. The cross-day spike-behavior decoding R^2^ value of MLP was 0.366, and the PCC of behavior-spike prediation was 0.349. The R^2^ value of ResNet+UNet was 0.390, and the PCC was 0.112. However, their performance in the cross-day task was still limited by the insufficient ability to adapt to the dynamic changes of neural signals.

In contrast, our model demonstrated strong generalization capabilities. It achieved an R^2^ of 0.581 for spike-behavior decoding and a PCC of 0.440 for behavior-spike prediction. This performance gain is attributed to the model's contrastive pretraining strategy, which aligns neural and behavioral representations across multiple sessions, and its use of hybrid SNN-ANN to extract event-driven and sequential features separately. It is worth noting that our model also maintains the advantage of keeping the same architecture in cross-day tasks. Through one training session and subsequent fine-tuning, bidirectional decoding functionality can be achieved. There is no need to modify the model structure for different tasks. In contrast, traditional methods and other deep learning models often require architectural adjustments and re-training across different tasks, which reduces the efficiency and universality of the system.

### 4.4 Case analysis

#### 4.4.1 Decoding task

**Figure 5:**
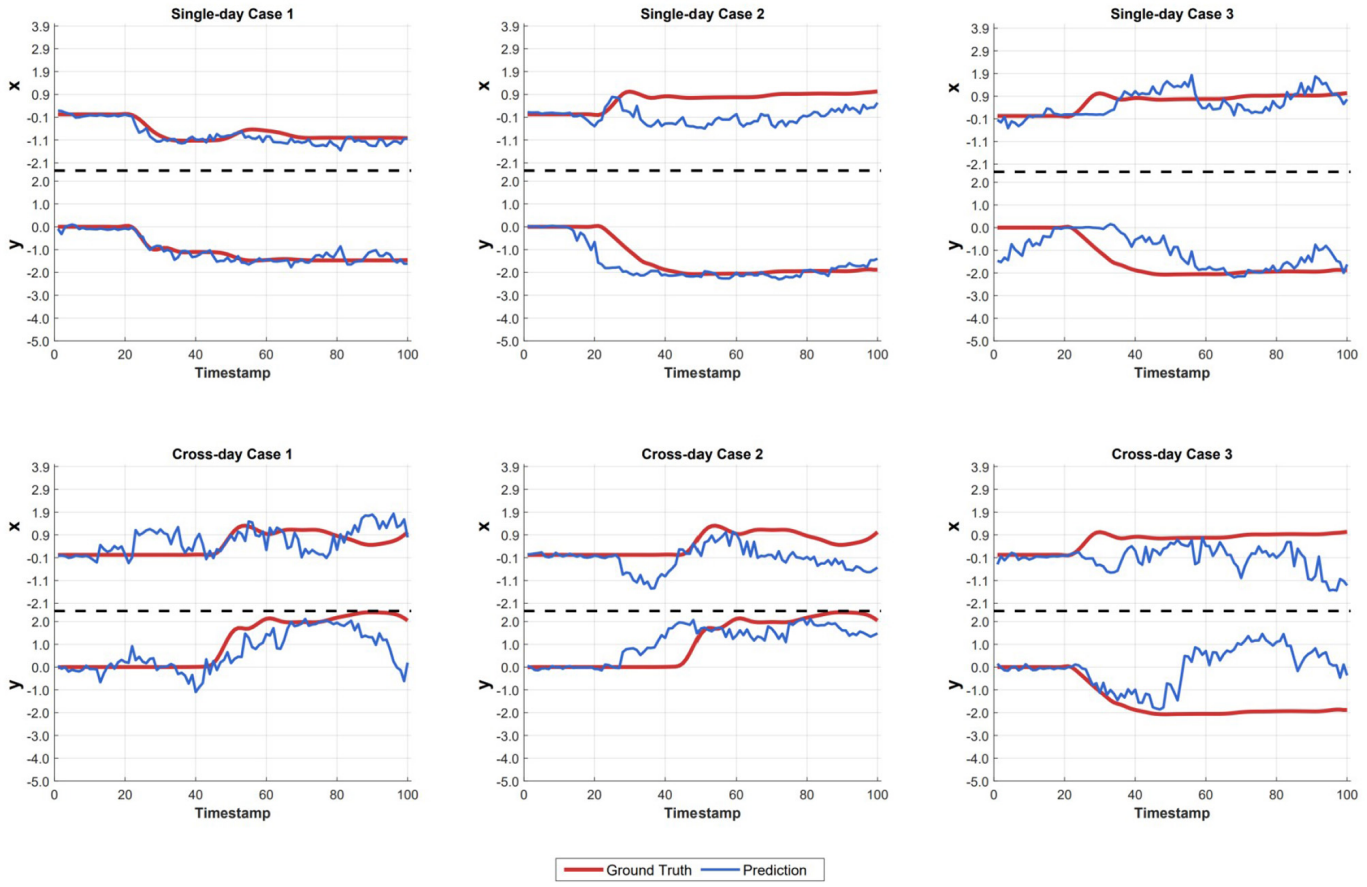
Spike-to-behavior case analysis. We present the results of three single-day and three cross-day case analyses. The upper part and lower part of each figure means the change of x and y axis of behavior decoding result, the red line means the ground truth while the blue line means the result of our prediction.

To visualize the model's decoding performance, we plot the decoding outcome graphs in Figure 5. We present the results of three single-day decoding cases and three cross-day decoding cases, with data selected randomly. In each subplot, the upper section shows the variation along the x-axis, while the lower section displays the variation along the y-axis. The red and blue lines represent the ground truth and prediction, respectively. We can clearly observe that in the single-day decoding results, the predicted values align closely with the actual values. To demonstrate the advantages of our model, in addition to the single-day results, we have also randomly selected three cross-day result plots. These include cases with both strong and relatively weaker fitting performance. We can find that our model can roughly depict the movement trajectory of the macaque. Nevertheless, the predicted trajectories confirm the effectiveness of our model in cross-day behavioral decoding tasks.

#### 4.4.2 Simulation task

**Figure 6:**
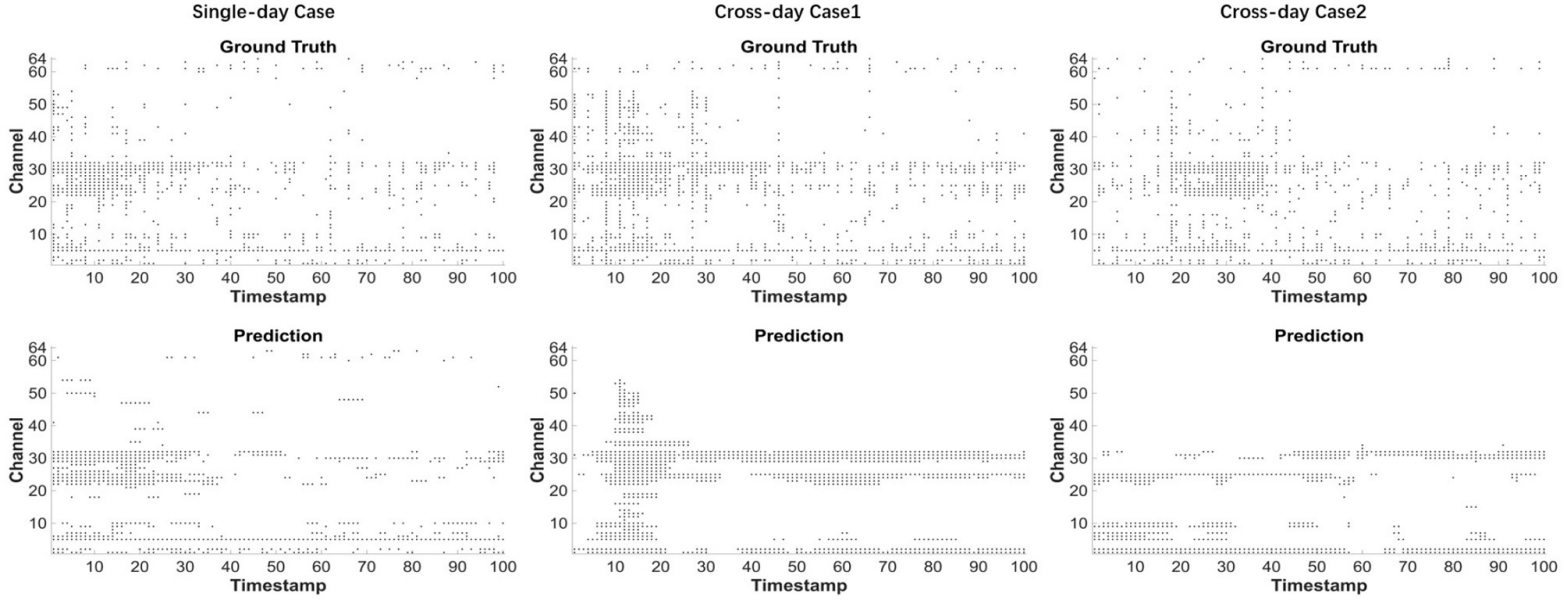
Behavior-to-spike case analysis. We display results from three single-day and three cross-day experiments. In each figure, the upper subplot depicts the ground truth while the lower subplot shows the performance of prediction result of our figure. X axis means the 100 time points while the y axis means 64 channels.

We present the results of behavioral spike simulation in Figure 6, where the leftmost plot shows the single-day simulation results, while the middle and right plots display cross-day results. The upper and lower subplots represent the ground truth and prediction, respectively, with the x-axis representing timestamps and the y-axis representing channel numbers, in a 100 × 64 dimension. In the single-day results, we observe that the model's predictions closely align with the ground truth across most channels and timestamps, demonstrating the model's high efficiency in single-day tasks. In the two cross-day results, the middle plot shows strong simulation performance in channels 20 to 35 and 1 to 10, but fails to capture spike activity in channels 55 to 64. In the right plot, the simulation achieves better results in channels 1 to 10, while partially reproducing neural activity in channels 20 to 35.

**Figure 7:**
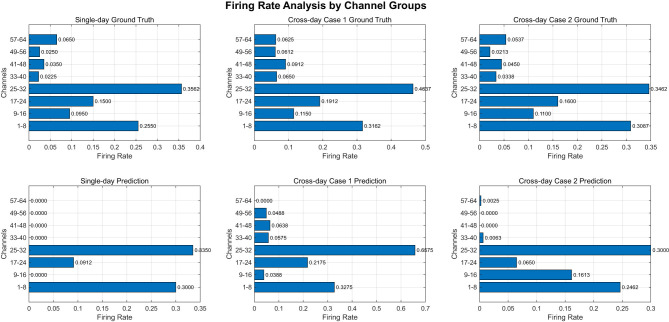
Channel comparison between ground truth and prediction. We compare the firing rates of ground truth and predicted neural activity across different channels, illustrating model performance for both single-day and cross-day scenarios. In each figure, 64 channels were split into eight groups while each group contains 8 channels.

To further compare the firing rate of different channels, we split the 64 channels of each trial into several groups. Every group contains 8 channels, the figure can be shown as Figure 7. In each plot, x axis means the average firing rate of different channels. The equation can be shown as Equation 20.


$$\begin{array}{l} \text{FR}\left[i\right]=\frac{1}{N}\overset{N}{\underset{t=1}{\sum }}{𝕀}_{\left\{S\left[i,t\right]\geq 1\right\}}. \end{array}$$ (20)

In this equation, i means the channel index while t means the value of t-th sampling points in i-th channel. The results reveal consistent firing rate patterns across both ground truth and prediction data. Specifically, channels 1–8 and 25–32 exhibit significantly higher firing rates in all experimental conditions, whereas channels 33–64 show substantially lower firing rates in the ground truth data and even more attenuated activity in the predictions. This differential pattern suggests that channels 1–8 and 25–32 may be more strongly correlated with macaque behavioral outputs while 33–64 shows little relationship with macaque behavior cross different days. We suggest that during cross-day training, our model effectively captures the dynamics of high-frequency neural activity but shows limitations in simulating channels with weak neuronal firing. This indicates that while some neurons may lack cross-day consistency or task relevance, our model successfully identifies and learns the simulation patterns of behaviorally-relevant neurons. These findings collectively demonstrate our model's superior capability in cross-day neural decoding tasks.

## 5 Conclusion

This work introduces an innovative network architecture that synergistically combines the advantages of hybrid SNN-ANN framework and contrastive learning. The proposed model simultaneously handles two key tasks: decoding behavior from neural spikes and simulating spikes from behavioral data. It achieves comparable performance under both single-day and cross-day training settings compared to existing baseline models. SNNs effectively extract features from neural spikes, while ANNs encode information from macaque behavior. The UNet architecture and contrastive learning collaboratively map both types of data into a shared latent space during training. Additional analysis of simulation outputs revealed that while strong inter-day consistency emerged in behaviorally relevant neural channels, others showed no detectable spike activity, suggesting that the model selectively captures task-critical neural representations. These findings highlight the broad utility and robustness of the proposed framework for neural decoding and simulation applications.

## Data Availability

The original contributions presented in the study are included in the article/supplementary material, further inquiries can be directed to the corresponding author.

## Footnotes

1
https://www.braindatacenter.cn/datacenter/web/#/home
